# Reply 2: NICE guidelines on drugs for colorectal cancer

**DOI:** 10.1038/sj.bjc.6600853

**Published:** 2003-04-01

**Authors:** P J Ross, D Cunningham

**Affiliations:** 1 Royal Marsden Hospital, London, UK; 2 Department of Medicine, Royal Marsden Hospital, Downs Road, Sutton, Surrey SM2 5PT, UK

**Sir**,

Drs Mason, Johnson and Rudd suggest that criticism of the guidance issued by the National Institute for Clinical Excellence (NICE) regarding the use of irinotecan and oxaliplatin in the treatment of patients with advanced colorectal cancer may have implications for the FOCUS trial (Cunningham *et al*, 2002; Mason *et al*, 2002; NICE, 2002; Saunders and Valle, 2002). However, the reason for criticism of the NICE guidance is due to the overwhelming evidence that access to the three drugs 5-fluorouracil, irinotecan and oxaliplatin is the optimal therapeutic approach to metastatic colorectal cancer. Subsequent to this guidance entry into the revised FOCUS protocol is the only way that patients in the UK can access all these agents. Therefore, patients not participating in the FOCUS trial will receive suboptimal therapy. Although the median survival for FOCUS of 16 months is encouraging, and certainly a major step forward over CRO6 with a median survival of approximately 10 months (Maughan *et al*, 2002), it remains significantly less than that for patients treated with all three agents in randomised trials such as N9741 (Goldberg *et al*, 2002) and the Tournigand trial (Tournigand *et al*, 2001) with median survivals of 18.6 months (data presented at the American Society of Clinical Oncology, Orlando, 2002) and approximately 21 months (data presented at the American Society of Clinical Oncology, San Francisco, 2001).

While recognising there may be merit in fine-tuning the sequence of use of these three agents (combinations *vs* monotherapy), the crucial point is that all patients should have access to these three drugs.
